# Household composition and child health in Botswana

**DOI:** 10.1186/s12889-019-7963-y

**Published:** 2019-12-03

**Authors:** Oleosi Ntshebe, Andrew Amos Channon, Victoria Hosegood

**Affiliations:** 1 Department of Population Studies, University of Botswana, Corner of Notwane and Mobuto Rd, Private Bag 705, Gaborone, Botswana; 2 Social Statistics and Demography, University of Southampton, Southampton, UK

**Keywords:** Household composition, Children, Stunting, Diarrhoea, Long-term health, Short-term health, LMICs, Index child

## Abstract

**Background:**

There is a general lack of research on children’s household experiences and child health outcomes in low- and middle-income countries (LMICs). This study examines the relationship between household composition, stunting and diarrhoea prevalence among children younger than 5 years of age in Botswana.

**Methods:**

The analysis uses data from the 2007 Botswana Family Health Survey (BFHS) and multilevel logistic regression models.

**Results:**

The findings indicate that stunting varies by whom the child lives with. Stunting is higher among children living with no parents compared to those living with both parents. Stunting is also high among children living with unrelated household members. Similarly, children in households with a mother-only and with a grandparent present, have a higher level of stunting compared to those living with both parents. Conversely, living with an aunt and living with other relatives, protects against stunting. The findings on diarrhoea prevalence show that children living in mother-only households and those living with no parents are less likely to have diarrhoea than those living with both parents. Also, across all households, those who are more affluent have lower rates of child stunting and diarrhoea than those which are more deprived. Finally, the findings show a clustering effect at the household level for both stunting and diarrhoea prevalence.

**Conclusions:**

These findings suggest that policies and programs aimed at reducing stunting and diarrhoea may work best if they target households and other adults co-residing in homes with children besides biological parents. Further, children who live in poorer households deserve special attention.

## Background

There is a general lack of research on child health outcomes such as stunting and diarrhoea prevalence and its relationship with household composition in low and middle-income countries (LMICs). Most of the research on family and household influences on child health comes from the United States of America (USA), the United Kingdom and other higher-income countries. This analysis aims to find out whether child health, as measured by stunting and diarrhoea prevalence among children less than 5 years of age in Botswana, is associated with the composition of the household. These two child health conditions have been chosen to represent both long-term and short-term health implications. Stunting develops over a long period, indicating a lack of sufficient nutrition. It is defined as low height for the child’s age [1, 2]. Conversely, diarrhoea is a short-term indicator of poor health and is related to poor environmental conditions [3].

The two-child health outcomes investigated in this manuscript represent significant health problems for children throughout LMICs. UNICEF [4] shows that stunting contributes to more than a third of deaths among children less than 5 years of age, and its prevalence is highest in sub-Saharan Africa (40.0% of the world’s cases) and South Asia (39.0% of stunted children in the world). The same report also states that diarrhoeal diseases remain a leading killer of young children contributing to 1% of world’s deaths during the neonatal period and to 10% of the deaths in the post-neonatal period [4]. Recent estimates also show that together stunting and diarrhoeal disease-account for almost half of the world’s child deaths [5].

Stunting and diarrhoea are also widespread among children in Botswana. According to official data, 26% of children under 5 years were stunted in 1993. The corresponding figure for 2000 was 23% [6, 7]. By 2007, the level of stunting among children had increased to 29.9% [8]. Data on diarrhoea in Botswana shows that its prevalence was about 20% among children under 5 years in 2004 [9].

The prevalence of stunting and diarrhoea is related to a broad range of factors. The causes of stunting include poor household food security, inadequate care, poor access to health services and unequal distribution in socio-economic resources [10–12]. Poor socio-economic conditions, poor breastfeeding practice, and increased risks of persistent illness are associated with diarrhoea prevalence [13].

Another important factor for child health outcomes is the role played by the child’s family and household members. For example, past studies of family structure in high-income countries (HICs) highlight the importance of parental time, economic and social resources for child well-being [14, 15], and familial stability [16] on health. Other studies show that households provide a physical place that supports childrearing, procreation, consumption and economic production [17–19]. Families and homes also provide resources to identify and manage child health [20, 21].

While most of the studies conducted on family and child well-being in HICs provide a good starting point for understanding the role of the family and household environment on child well-being, it remains critical to explore the role of households on child health in the LMIC context. Household environments in LMICs can be very different from those that are standard in HICs, with associated differences in the context of child-rearing, which might result in differing results between HICs and LMICs contexts. For example, in HICs, the focus has tended to be on the influence of one- or two-parent families, with less consideration of the other adults in the household [22], which may be necessary for LMICs.

Research in LMICs indicates that households are larger and more complex than those in HICs concerning membership. Children in LMICs are raised in homes with higher levels of non-parental residence, and with a higher proportion of other household members [23–26] outside of the traditional nuclear family. In the case of Southern Africa, children are likely to live in multi-generational households and along with grandparents [27], as well as live apart from their mothers [26].

Moreover, research to date has not fully explored household relationships from the view of the child; that is by establishing their relationship to everyone else in the household from their perspective. Earlier work has defined household composition as relationships of household members only to the household head [28–30], or kin [31, 32]. However, this approach is limited since it does not show the relationship of each member to the rest of the household members. Additionally, this traditional perspective of household membership is problematic when focusing on children when the relation of the child to the broader household membership is of interest and not only the relationship between the children to the head of the household. Thus, the current analysis constitutes a first step in using more expanded categories of the household composition to understand its impact on child health.

The purpose of this study is to examine the types of household composition as operationalised from the relationship of the child to other household members. It will then extend this to assess if child health outcomes vary by the household composition. The main focus will be on whether the child lives with both parents, only one parent, or no parents at all, as well as the types of other individuals living in the household (such as grandparents, aunts, uncles, other relatives or unrelated household members). Lastly, the study examines the extent to which different types of household members moderate the relationship between living with parent(s) and child health outcomes.

## Methods

The analysis uses data from a nationally representative survey, the 2007 Botswana Family Health Survey (BFHS). The 2007 BFHS contains information on maternal and child health outcomes as well as data on the demographics, family background and household living conditions for children aged less than 5 years. The sampling design for the 2007 BFHS involved two stages. The first stage of sampling involved a selection of enumeration areas (EAs) proportional to the number of households in the EA. The second stage of sampling involved a systematic selection of 7841 occupied households from the selected EAs [8]. Within the sampled households, all the 5021 eligible women aged 15–49 were interviewed, and detailed information about their households and all their children aged less than 5 years were collected [8].

The 2007 BFHS data has a hierarchical structure in which 2531 children are nested within 1804 households and 298 enumeration areas for stunting prevalence. For diarrhoea prevalence, 2713 children are nested in 1892 households and within 298 EAs. The data consists of 2531 children for stunting, excluding 184 children who had missing data on the age variable and height for age z scores (HAZ) and had implausible values outside the range − 6/+ 6 HAZ. For diarrhoea prevalence, the count is 2713 children as only two children had missing data on the outcome.

### Variables

#### Dependent variables

Two outcomes are of interest: stunting and diarrhoea prevalence. Stunting is an indicator of long-term growth prospects and prior adverse conditions that affect a child’s nutritional status [1, 2, 12, 33]. Stunting is defined using height-for-age z-scores (HAZ). Children with z-scores less than two standard deviations (< −2SD) below the median on height-for-age in comparison to the World Health Organization international reference standard are considered stunted [1, 2].

Statistics Botswana enumerators collected anthropometric data for all sampled children [8]. The study uses anthropometric indices of length for children younger than 24 months and height measurements for those 24 months and older [1]. Children aged less than 24 months had their length in centimetres recorded from using a measuring board — children aged 24 months and over had their height measured in centimetres standing upright. The response rate for anthropometric measurements was 98.3% [8].

Diarrhoea prevalence is a proxy for short-term poor health and poor environmental conditions [3]. Diarrhoea was determined by asking mothers if their children experienced loose or watery stools, or blood in the stool, in the last two weeks preceding the survey. Both the child health outcomes are coded one (1) for the presence of the illness and zero (0) for its absence.

#### Main explanatory variable

The primary independent variable is household composition which consists of two separate indicators: parental presence and the presence of other types of household members. Parental presence is a four-categorical variable related to whether the child lives with both parents, only the mother, only the father or no parents. The types of other household members that could be living in the household are grandparents, aunts, uncles, other relatives and members that are unrelated. The variables indicating the presence of other household members have been coded as dummy variables.

#### Control variables

The control variables include both household context and child variables. The household contextual variables are household wealth, household size, number of children aged less than 5 years in the household, the region of residence, and place of residence. A household wealth index was calculated from the presence of household assets (e.g. radio, television, motor vehicle, tractor and cattle etc), the material of construction of the main house (wall, roof, and floor materials), types of fuels used in the home, source of drinking water and type of toilet facility. The household wealth score was obtained using principal components analysis (PCA) following the standard methodology [34] and divided into five equal groups of 20% of household (quintiles) at the national level. Thus, in the dataset, the household wealth index variable has five categories (poorest, second, middle, fourth and richest). Household size has three groups: 2–3 persons, 4–6 persons and 7 and more persons.

The number of children aged less than 5 years has four categories: 1 child, 2 children, 3–4 children and 5–6 children. Region has five categories: North, South, West, East/North East and Central. Place of residence is also categorical: 1 = City/Town, 2 = Urban village and 3 = Rural. Other variables are the age of the child measured in months and sex of the child (0 = Male and 1 = Female).

### Data analysis

Data analysis was conducted in *Stata version 13* and *MLwiN*. Only households with at least two persons (including a child aged 0–59 months) were analysed. The results are presented using descriptive statistics and multilevel logistic regression. Tests for the association between categorical variables were Pearson’s Chi-Square test and Fisher’s exact test [35]. The 95% confidence intervals (CI) for all bivariate estimates are shown.

Multilevel modelling is carried out to account for clustering of children within households and within enumeration areas. The results of the multilevel logistic regression modelling are presented as odds ratios (OR) along with the 95% CI and the *p*-values. Three models were estimated for multilevel logistic regression modelling. The first model is basic (model 1), and it contains all level 1 explanatory variables and level 2 explanatory variables. The second model (model 2) has all the explanatory variables in model 1 and the interaction between the parent presence variable and the presence of other household members. The third model (model 3) includes variables in model 2 and a random slope on household wealth (5th quintile) and age of the child. A random slope on the top quintile of wealth and age of the child for stunting and diarrhoea prevalence were not significant. Hence, the modelling of random coefficients was not pursued.

Testing for multicollinearity between the set of predictor variables was also conducted [36]. There was no evidence of collinearity. A test for the interactions between living with parent(s) and other types of household members found that only the interaction between mother and grandparent presence in the household was significant.

The estimations for the multilevel logistic models were conducted in MLwiN using the 2nd order predictive quasi-likelihood method (PQL2). The PQL2 is preferred, as Maximum likelihood (MQL1) may produce estimates which are biased downwards [37]. At each stage of modelling the models were evaluated using the Wald test [38, 39]. The variance partition coefficient (VPC) or how much of the total variance is attributable to a level 3 and 2 was also determined using an approach specified by [40, 41]. The general form of the multilevel logistic regression model used in the analysis and the equations for the estimation of the VPCs are available upon request from the authors.

## Results

### Descriptive analysis

Descriptive analysis was conducted to show sample characteristics by age, household size, types of stunting, and household membership. The mean age of the children in the data was found to be 28.5 months (SE = 0.35), and the mean household size was found to be 7.03 (SE = 0.07) persons. Household size is large because many households with children include not only biological parents but relatives and other unrelated members [23].

About the child health outcomes, the results show an overall prevalence of stunting at 29.9% (95% CI 28.0, 31.9%) among children aged less than 5 years. The stunted children were then classified as to the degree of stunting [1, 2]. The results show that 13.4% (95% CI 12.1, 14.9%) of the children in the study were severely stunted (≥ − 6SD and < −3SD), 16.7% (95% CI 15.1,18.4%) of the children were moderately stunted (≥ − 3SD and < −2SD), 25.9% (95%CI 24.1,27.8%) of the children were mildly stunted (≥ − 2SD and < −1SD), and the remaining 44.0% were better nourished (95% CI 41.9,46.1%). On the other hand, the results of diarrhoea prevalence were 18.0% (95% CI 16.5, 19.6%) among children aged 0–59 months.

A classification of the parenting household types shows that a child either lives with a mother only, fathers only, both parents, and no biological parents. The largest share of children come from households with both parents (44.1%), 25.5% come from mother-only families, 14.1% come from father only families, and the rest are from no parent households (16.3%). About co-residence with other household members, the results show that 20.0% of the children live with grandparents, 31.1% live with uncles, 33.1% live with aunts, 10.1 live with other relatives, and 9.1% live with unrelated members.

### Bivariate analysis

**Fig. 1:**
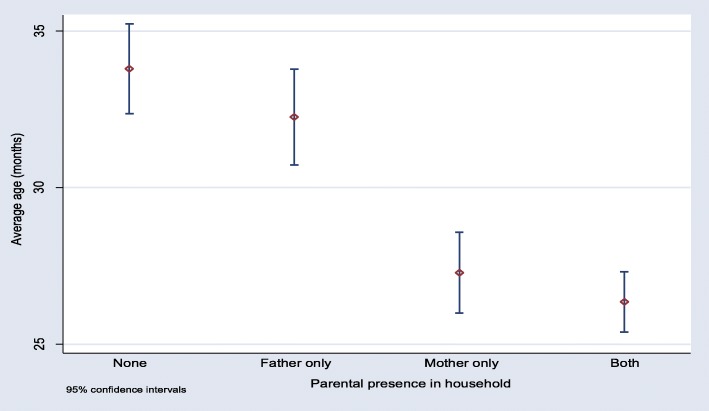
Presence of a parent (s) in a household and the child’s age

A further descriptive of the household circumstances of children of various ages is shown in Fig. 1. Figure 1 shows that younger children are likely to live with only their mother and with both parents than older children. While living with neither parent and only with father is more likely for older children than younger children. The association between a child’s age, co-residence with other household members and with household wealth was also examined. However, the results showed no association between the variables (see Additional file 1).

Table 1 examines child health outcomes by household and child characteristics. Household wealth is significantly associated with both stunting and diarrhoea. Poorer households (1st-4th quintiles) have higher disease prevalence than those in the wealthiest households (5th quintile). The findings on household wealth, stunting and diarrhoea prevalence are not surprising, and they are consistent with prior research evidence. Several studies indicate that wealthier households confer better nutrition and economic and social resources for better child health care than poorer households [42–44].

**Table 1:** Per cent distribution of child health outcomes by household and child characteristics, BFHS 2007

Characteristic	Stunted (below −2 SD height/age)					Diarrhoea in the last two weeks before the survey				
	%	Total	N	% missing	95% CI	%	Total	N	% missing	95% CI
Household size (persons)										
2–3	27.3	263	273	3.7	21.9,33.4	17.0	273	273	0.0	12.7,22.4
4–6	27.7	1004	1094	8.2	24.6,30.9	16.3	1092	1094	0.2	14.1,18.8
7+	32.4	1206	1294	6.8	29.7,35.2	19.6	1293	1294	0.1	17.4,22.0
Household wealth										
Poorest	33.8	687	740	7.1	30.1,37.6	22.3	736	740	0.5	19.2,25.8
Second	35.1	719	771	6.8	31.6,38.8	18.2	771	771	0.0	15.6,21.2
Middle	27.7	398	429	7.3	23.3,32.7	18.1	429	429	0.0	14.6,22.3
Fourth	25.3	361	390	7.6	20.0,31.5	16.9	390	390	0.0	13.2,21.2
Richest	17.6	309	332	6.7	13.6,22.6	8.9	332	332	0.0	6.1,12.8
Region^a^										
North	26.2	280	302	7.3	21.1,32.1	21.3	302	302	0.0	16.6,26.9
South	25.3	718	778	7.7	22.0,29.0	16.3	778	778	0.0	13.7,19.3
West	33.0	442	486	8.9	28.7,37.6	21.6	484	486	0.3	18.1,25.6
East & North East	33.8	256	271	5.6	26.9,41.5	14.3	271	271	0.0	10.6,19.1
Central	32.5	779	826	5.7	29.2,36.1	17.5	824	826	0.3	14.9,20.4
Residence										
City/Town	25.6	411	441	6.8	20.7,31.1	15.8	441	441	0.0	12.6,19.7
Urban village	27.8	822	879	6.5	24.8,31.1	17.3	879	879	0.0	14.9,20.1
Rural	32.8	1241	1342	7.5	30.0,35.6	19.1	1339	1342	0.3	16.9,21.5
Number of children under five years in a household										
1–2	29.1	1975	2129	7.2	26.9,31.3	17.1	2125	2129	0.2	15.5,18.9
3–6	33.3	499	533	6.4	29.2,37.8	21.5	533	533	0.0	18.0,25.4
Child’s age group (months)										
0–5	23.9	215	238	9.7	18.3,30.7	16.8	238	238	0.0	12.3,22.5
6–11	28.7	271	299	9.6	23.4,34.7	28.3	299	299	0.0	23.2,33.9
12–23	43.4	539	577	6.7	38.7,48.3	29.1	577	577	0.0	25.1,33.3
24–35	35.7	534	572	6.6	31.6,40.0	17.7	572	572	0.0	14.7,21.2
36–47	24.0	487	526	7.3	20.3,28.2	9.1	524	526	0.3	6.8,11.9
48–60	16.4	429	450	4.8	13.0,20.5	8.3	448	450	0.5	5.9,11.4
Child’s sex										
Female	27.3	1233	1324	6.9	24.6,30.2	19.0	1322	1324	0.2	16.8,21.4
Male	32.6	1241	1338	7.3	29.9,35.4	17.0	1336	1338	0.1	14.9,19.2
Grand Total	29.9	2474	2662	7.1	28.0,31.9	18.0	2658	2662	0.1	16.5,19.6

Total = all children with and without stunting/diarrhoea. N = All children, including those with missing data. ^a^Region is coded from all the districts in Botswana. North includes Ngamiland East, Ngamiland West, Ngamiland Chobe, Kgalagadi North districts. South includes Gaborone, Lobatse, Jwaneng, South East, Kweneng East, Kgatleng districts. West includes Southern Ngwaketse, Southern Borolong, Southern Ngwaketse West, Kgalagadi South, Kweneng West, Ghanzi districts. East and North East include Francistown, Selebi Phikwe, and North East districts. Central covers Orapa, Sowa, Serowe/Palapye, Mahalapye, Bobirwa and Tutume districts

Table 1 also shows child health outcomes by child’s age group and sex. Stunting and diarrhoea are high among children aged 12–23 months. Moreover, for both the health outcomes, the prevalence of the illness is lowest in the age group 48–60 months. About sex, the prevalence of stunting is higher for males (32.6%) than females (27.3%). On the other hand, for diarrhoea, there is not a big difference in the prevalence between males (19.0%) and females (17.0%). Further, Table 1 shows that region and residence matter for stunting while household size and the number of children less than 5 years of age in a household are important for diarrhoea prevalence.

Table 2 presents the bivariate relationship between child health outcomes and the presence of biological parents and other household members. Table 2 shows that stunting is associated with living with neither parent (32.6%), unrelated household members (31.1%), and living only with a father (30.8%). Table 2 also shows that diarrhoea is more likely for children living in households where only the father is present (21.1%), and those living with a grandparent (20.6%).

**Table 2:** Per cent distribution of child health outcomes by the presence of biological parents and other household members, BFHS 2007

	Stunted (below −2 SD height/age)					Diarrhoea in the last two weeks before the survey				
	%	Total	N	% miss.	95% CI	%	Total	N	% miss.	95% CI
Children by the presence of parents and other household members										
Mother only^a^	30.4	631	680	7.2	26.8,34.4	15.5	680	680	0.0	12.8,18.5
Father only^b^	30.8	349	375	7.1	25.9,36.1	21.1	373	375	0.6	17.1,25.8
Both parents	28.4	1093	1174	6.9	25.5,31.5	20.1	1174	1174	0.0	17.7,22.7
No parents	32.6	401	433	7.3	28.1,37.5	13.5	431	433	0.3	10.5,17.2
Mother present^c^	29.1	1724	1853	7.0	26.8,31.6	18.4	1853	1853	0.0	16.6,20.4
Father present^d^	29.0	1442	1549	6.9	26.4,31.6	20.3	1547	1549	0.1	18.3,22.6
Grandparent	29.5	505	531	5.0	25.5,33.8	20.6	531	531	0.0	17.3,24.5
Uncle	30.9	779	825	5.7	27.6,34.5	19.7	823	825	0.3	17.0,22.7
Aunt	29.9	828	881	6.1	26.7,33.3	18.6	879	881	0.2	16.0,21.4
Other relatives	27.3	252	268	6.1	20.5,35.3	14.8	268	268	6.1	10.7,20.0
Not related member	31.1	229	243	5.8	24.9,38.0	14.5	243	243	0.0	10.5,19.7
Grand Total	29.9	2474	2662	7.1	28.0,31.9	18.0	2658	2662	0.1	16.5,19.6

Total =all children with and without stunting/diarrhoea. N = All children, including those with missing data. ^a^only the mother of the child is listed in the household, and not the father.^b^only the father of the child is in the household, and not the mother. ^c^determined from the type of household head and whether the mother is present in a household; MH + MP, FH + MP, GH + MP, UH + MP, AH + MP, ORH + MP, NRH + MP. ^d^determined from the type of household head and whether the father is present in the household; MH + FP, FH + FP, GH + FP, UH + FP, AH + FP, ORH + FP, NRH + FP. *M* Mother, *H* Household head, *F* Father, *G* Grandparent, *U* Uncle, *A* Aunt, *OR* Other relatives, *NR* Not related member, and *P* Present

Further analysis of the association between child health outcomes and classification of household member composition as independent and mutual events was also carried out. Details of such bivariate analysis are available in Appendix (see Additional files 2 and 3).

### Multivariate analysis

Table 3 shows that several variables are statistically associated with stunting. These variables include living with no parents, living with an aunt, other relatives and unrelated household members, household wealth, region, sex of the child and age of the child. None of the parental presence variables; defined as a child lives with a mother or father, compared with a child living with two parents, is statistically significant at 5% level of significance, although the association is in the expected direction (Table 3).

**Table 3:** Fitted random intercept models for child health outcomes, BFHS 2007

	Stunting						Diarrhoea					
				95% CI						95% CI		
Variable	Coef.	S.E.	OR	Lower	Upper	p-value	Coef.	S.E.	OR	Lower	Upper	*p*-value
Fixed Part												
Intercept	−1.791	0.375	0.167	0.080	0.348	***	−0.284	0.434	0.753	0.322	1.762	
Parent (s) in a household												
Both parents (ref.)												
Mother only	0.055	0.154	1.057	0.781	1.429		−0.431	0.176	0.650	0.460	0.918	**
Father only	0.000	0.200	1.000	0.676	1.480		0.287	0.191	1.332	0.916	1.937	
No parents	0.419	0.181	1.520	1.066	2.168	**	−0.480	0.215	0.619	0.406	0.943	**
Other household members												
Grandparent	−0.253	0.241	0.776	0.484	1.245		0.173	0.168	1.189	0.855	1.652	
Aunt	−0.297	0.132	0.743	0.574	0.962	**	0.103	0.157	1.108	0.815	1.508	
Uncle	0.147	0.160	1.158	0.847	1.585		−0.305	0.187	0.737	0.511	1.063	
Other relatives	−0.348	0.191	0.706	0.486	1.027	*	−0.092	0.229	0.912	0.582	1.429	
not related	0.367	0.182	1.443	1.010	2.062	**	−0.265	0.237	0.767	0.482	1.221	
Household wealth												
Poorest (ref.)												
Second	0.100	0.151	1.105	0.822	1.486		−0.136	0.181	0.873	0.612	1.245	
Middle	−0.253	0.194	0.776	0.531	1.136		−0.271	0.232	0.763	0.484	1.202	
Fourth	−0.594	0.213	0.552	0.364	0.838	***	−0.394	0.248	0.674	0.415	1.096	
Richest	−0.917	0.248	0.400	0.246	0.650	***	−1.367	0.321	0.255	0.136	0.478	***
Household size (persons)												
2–3 persons (ref.)												
4–6 persons	−0.069	0.193	0.933	0.639	1.362		−0.024	0.233	0.976	0.618	1.541	
7+ persons	0.063	0.219	1.065	0.693	1.636		−0.035	0.263	0.966	0.577	1.617	
Number of children aged less than five years												
1 (ref.)												
2	0.159	0.130	1.172	0.909	1.513		−0.103	0.156	0.902	0.664	1.225	
3–4	0.228	0.169	1.256	0.902	1.749		0.117	0.197	1.124	0.764	1.654	
5–6	0.770	0.516	2.160	0.786	5.938		−0.558	0.772	0.572	0.126	2.599	
Region												
North (ref.)												
South	0.156	0.224	1.169	0.753	1.813		−0.153	0.259	0.858	0.517	1.426	
West	0.343	0.217	1.409	0.921	2.156		0.136	0.246	1.146	0.707	1.856	
East & North East	0.521	0.277	1.684	0.978	2.898	*	−0.430	0.335	0.651	0.337	1.254	
Central	0.281	0.201	1.324	0.893	1.964		−0.184	0.232	0.832	0.528	1.311	
Residence												
City/town (ref.)												
Urban Village	−0.004	0.222	0.996	0.645	1.539		−0.440	0.258	0.644	0.388	1.068	*
Rural	−0.018	0.228	0.982	0.628	1.536		−0.496	0.271	0.609	0.358	1.036	*
Child’s sex												
Female (ref.)												
Male	0.360	0.103	1.433	1.171	1.754	***	−0.137	0.123	0.872	0.685	1.110	
Child’s age (months)												
Age	0.078	0.013	1.081	1.054	1.109	***	0.012	0.015	1.012	0.983	1.042	
Age^2	−0.002	0.000	0.998	0.998	0.998	***	−0.001	0.000	0.999	0.999	0.999	***
Interactions												
Both parents*grandparent (ref.)												
Motheronly*grandparent	0.694	0.366	2.002	0.977	4.102	*						
Fatheronly*grandparent	0.498	0.359	1.645	0.814	3.326							
Noparents*grandparent	−0.529	0.388	0.589	0.275	1.260							
Random Part												
Level 3: ea												
Cons/cons	0.127	0.073	1.135	0.984	1.310	*	0.150	0.100	1.162	0.955	1.413	
Level 2: HHId												
Cons/cons	0.703	0.161	2.020	1.473	2.769	***	0.922	0.225	2.514	1.618	3.908	***
Level 1: ChildId												
Bcons.1/bcons.1	1.000	0.000	2.718	2.718	2.718		1.000	0.000	2.718	2.718	2.718	
Statistics												
EA. level VPC (%)	3.08						3.44					
Household-level VPC (%)	17.06						21.14					
Units: EA	298						298					
Units: HHId	1804						1892					
Units: ChildId	2531						2713					

Variable ‘parent (s) in a household’ is a categorical variable with four levels, and both parents is the reference category. The variables ‘other house members’ are each dummy (1 for the presence of a household member and 0 for the absence of the household member). *ref*. reference category. *Coef*. Coefficient, *S.E* Standard error, *EA* Enumeration area, *VPC* Variance partition coefficient; cons/cons = residual variance at a particular level; *OR* odds ratio; 95% CI = 95% confidence interval

Legend: * *p* < .1; ** *p* < .05; *** *p* < .01

Table 3 also shows that children living in affluent households (5th quintile) have a 60% decrease in the odds of being stunted compared to those in the poorest households (1st quintile). Conversely children living in households in the fourth quintile have 45% lower odds of being stunted [(1–0.55)*100 = 45.0%], compared to those in the poorest households.

Further, Table 3 shows that concerning the age of the child, there is an 8% increase in the odds of being stunted with each additional month (OR = 1.08; 95% CI 1.05–1.11). By sex, males have 43% greater odds of being stunted than female children (OR = 1.43; 95% CI 1.17–1.75)*.*

### Factors associated with diarrhoea prevalence

About diarrhoea prevalence, there is an indication in Table 3 that living with a mother and living without parents is associated with a decrease in the odds of the illness, compared to living with both parents. For example, the odds ratio attached to the variables mother only (OR = 0.65; 95% CI 0.46–0.92) and no parents (OR = 0.62; 95% CI 0.41–0.94) are significant at *p* < 0.05 (see Table 3). None of the types of other living in the household, including a grandparent, aunt, uncle, other relatives or unrelated household members is significantly associated with diarrhoea prevalence.

Other factors associated with diarrhoea prevalence are household wealth and residence. However, place of residence is weakly associated (*p* < 0.1) with diarrhoea prevalence. Children living in affluent households are less likely to have diarrhoea compared to those in the poorest households (OR = 0.26; 95% CI 0.14–0.48).

Table 3 also displays the results of random effects. The results in Table 3 show a significantly larger and sizeable clustering effect at a household level than at the enumeration area level. The results indicate that for stunting, 17.1% of the unexplained variation in the disease lies at the household level (*p* < 0.01) and 3.1% of the difference lies at the enumeration area level (*p* < 0.1). For diarrhoea prevalence, 21.1% of the overall variation in the disease is attributable to the household (*p* < 0.01), and 3.4% is due to enumeration area.

**Fig. 2:**
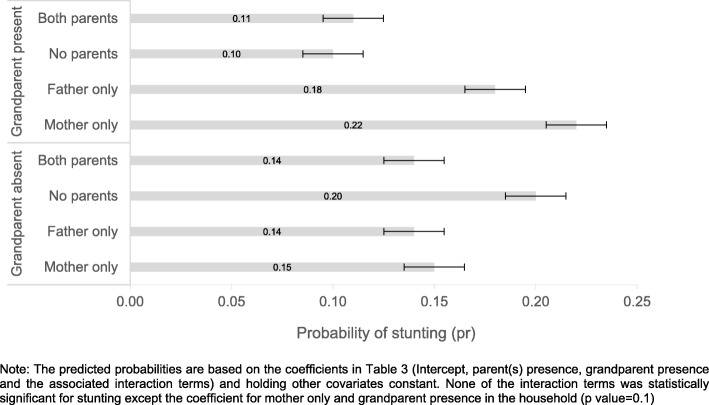
Graphical representation of the probability of stunting corresponding to the presence of a parent (s) and grandparent in the household

Lastly, Fig. 2 shows the probability of stunting by the presence of a grandparent and parents in the household. Figure 2 reveals a statistically significant difference between stunting for children living with a grandparent, and those living with no grandparent. However, the probability of being stunted is not different among children living with a grandparent and both parents and those living with no grandparent and both parents.

## Discussion

This manuscript examined household composition and child health using BFHS 2007 data. The descriptive results show that the range of actors involved in child-rearing in Botswana is extensive and much broader than biological parents. It includes grandparents, aunts, uncles, other relatives, and persons not related to the child. This finding is consistent with the results of an examination of children living arrangements in 19 developing countries, which have found that children in this context live in more extensive and diverse households than in high-income countries [26].

Several studies show that there are benefits for living in extended households for children. Evidence from the USA suggests that living with extended family for single parents provides opportunities for childcare and a safety net if the parent cannot afford childcare alone [45]. Similarly, studies in the developing countries show that living with extended household members enables access to socio-economic resources provided by adult household members such as grandparents and aunts [46–48]. Evidence also indicates that extended households offer opportunities to pooling of socio-economic resources to assist with child monitoring and supervision [23, 49]. However, larger households in developing countries also present competing demands for resources, which disadvantage children about insufficient food, inadequate attention and deficient health care [28].

The results from the multivariate analysis indicate that whether the child lives with none, one or two parents, as well as whom the parent lives with, it matters for stunting and diarrhoea prevalence. In particular, for stunting, living with neither parent increases the odds of stunting, compared to living with both parents. Also, the results show that stunting is more prevalent among children living with unrelated household members, and it is less among those children living with an aunt. The protective association of living with relatives on the risk of stunting suggests that kin may care more about the child than unrelated household members. A review of living arrangements of children from 19 developing countries shows that there is more commitment to childcare and provision of financial resources among children in households with parents and other related adults [26]. Research by Bronte-Tinkew and DeJong in Jamaica report that living with biological parents offer more care and provision of food resources that prevent child malnutrition [23].

Besides food resources and parental care, variation in access to health care is linked to the differences in child nutritional status. A study on child feeding practices and access to health services in Sierra Leone found household discrimination in food allocation and access to medical care toward foster children [50]. Furthermore evidence in Guinea-Bissau indicate that motherless children are often more malnourished than those living with their mothers, as they are placed in smaller and grandparent’s households, which are more likely to take a traditional approach to health care [51].

The findings from our study also confirm the results of other studies showing that household wealth, child’s age and sex are associated with stunting. About household wealth, the results show that those who are more affluent, regardless of who is in the household, do better in stunting and diarrhoea prevalence than those who are poorer. Other studies show that low household wealth is associated with increased risks for stunting [42], and higher rates of diarrhoea in infancy [43] from a lack of resources to ensure proper nutrition and good hygienic practices [23]. It may also be that richer in contrast to poorer households can offer opportunities for economic and social influences that promote and maintain good health [20, 21, 52].

Due to sex, male children in Botswana remain at a higher risk of stunting compared to females. This finding is as expected and consistent with prior research which indicates that stunting is more prevalent among male than female children [11, 23, 53, 54]. Since Batswana do not hold rigid notions and preference of children by sex, this finding is probably for biological and behavioural reasons. A longitudinal study of child development in Quebec by Côté, Blanchard [55] found that males are more prone to low birth weight, which is affected by maternal nutrition and health behaviours during pregnancy. Therefore, the finding on male vulnerability to stunting in Botswana necessitates continued care and careful monitoring of males in infancy and childhood.

By age, the findings in Botswana indicate that stunting is highest at 12–23 months. This result is not surprising given that the effect of stunting is long-term, cumulative, and more pronounced at 12–23 months [56, 57]. Some previous research finds that the lack of care and nourishment during early childhood is associated with higher risks of stunting among children aged between 6 and 23 months [58]. These findings suggest that household factors such as wealth and environmental condition are possible exogenous factors associated with low nutritional status. Accordingly, future research would benefit from considering these factors.

Our study also found that only one interaction between parental presence and grandparent presence showed a modifying effect on the relationship for stunting. The results showed that children who live with a mother and grandparent were slightly more stunted compared to those living with both parents. This finding is likely due to the strained coping capability of mother-only households to provide economic and food resources that are needed to prevent stunting. As reflected in other studies, mother-only families are much more likely to be poorer and not able to provide food and material needed for healthy child growth [14, 59], compared to living with both parents [29, 60].

A study by Mokomane, Baker [61] in rural and urban settings in Botswana shows that most of the single mothers who co-resided with their mothers were not employed. Thus, for mother-only households, co-residing with a grandmother may imply a lack of economic resources to maintain one’s household. On the other hand, two-parent households where grandparents co-reside may have enough financial resources to ensure proper child nutrition and care of extra members. Other studies suggest that co-residence with grandparents implies care of the grandparent [62, 63] and grandchildren [48].

Concerning diarrhoea prevalence in Botswana, the findings indicate that children living only with a mother and those living with neither parent are less likely to have diarrhoea compared to those living with both parents. This finding is contrary to expectation since it is often assumed that living with both parents is beneficial for child well-being compared with living with a single parent [15, 16, 64, 65]. 

The comparison of living with neither parent and living with both parents on diarrhoea prevalence may reflect the transitory movement of children with no parents into households that can provide for them. For example, a study on the orphanage and nutritional status of Luo children in Kenya indicates that orphans often end up in wealthier households than non-orphans [66]. Evidence from Botswana also shows that working or income-earning households are often the preferred choice for providing the basic needs of orphaned children in the short-term [67]. However, the cross-sectional nature of the data used in the study by Miller, Gruskin [67] and Zidron, Juma [66] do not allow an analysis of financial resources of the parent (s) and the nature and timing of fostering of children by extended family members on child health outcomes. This information would aid in interpreting the effects of no parents household on both long-term illnesses (stunting) and on the short-term disease (diarrhoea).

Finally, the results on clustering indicate that most of the variance in stunting and diarrhoea prevalence is attributed to differences between households than to enumeration areas. Thus, this finding echoes other studies, which show that households are shared contexts, and children within the same household are likely to have similar risks of time, social and economic resources for child nutritional status [68–71], as well as the use of health services [72, 73]. On the other hand, the small clustering effect at the EA level may imply the little impact of the geographical contextual effect on the two-child health outcomes. Also, a small clustering effect at the EA level may merely be an indication of the presence of other real EA effects which are not accounted for in the present analysis.

### Limitations

This study has some limitations. First, given the cross-sectional nature of the data, no causal effects are made. Instead, the findings provide information on a child’s experience of a household at a point in time. Longitudinal data would be necessary to illuminate the causal effects and influences, which led to the household composition, intra-household links and its implications for child health.

Second, mothers or caretakers reported information on the child. In particular, the measure for diarrhoea prevalence is provided by mothers without confirmation of a physician. Thus, the analysis might be affected by social desirability bias from the characteristics of the mother/caretaker such as her age, experience with childcare, education and social context she lives in.

Third, the dataset does not permit control for other variables such as mother’s age, marital status, and education. These variables were considered in the initial analysis but were dropped due to a high number of missing values on them. However, the afore-mentioned limitation was mitigated by the inclusion of the household wealth variable to capture the influence of the household environment and household resources on child health outcome.

Also, other factors not investigated here could be associated with either stunting and or diarrhoea. Potential factors include maternal health, maternal and child HIV status, breastfeeding practices, maternal nutrition, the duration of living in a particular household and the nature of childcare. Future analysis can include key variables not captured in the current study, to ensure that the associations between child health and household composition are not overstated.

However, despite these limitations, the study highlights the need to understand the relationship between child health and household composition in LMICs. The results are also satisfactory, and the data quality is adequate. Additional confidence in the findings is the fact that the data is nationally representative, and Statistics Botswana collected it. Statistics Botswana made sure that training of the interviewers was thorough, and data were checked for accuracy and completeness at fieldwork and entry stages.

## Conclusions

The results from this study provide a basis for enhanced efforts to improve child health across households in Botswana. The findings show that stunting is higher among children living with no parents compared to those living with both parents. Stunting is also high among children living with unrelated household members. Also, children living in mother-only households and with a grandparent present have a higher level of stunting compared to those living with both parents. Further, the results on long term illness indicate that living with an aunt and living with other relatives protects against stunting.

The analysis of diarrhoea prevalence indicates that the presence of a mother and the absence of both parents are more critical for reducing this short-term illness. However, it is not clear if this is the case in the long term. Across all households, the results on stunting and diarrhoea prevalence show that children living in a wealthy household are much more likely to be healthier than those living in a poor household. Moreover, the results indicate variability in child health outcomes across households. The findings show a clustering effect at the household level at 17.1% for stunting and 21.1% for diarrhoea prevalence.

Finally, the research demonstrates a nuanced way of investigating household relationships from a child’s view, rather than establishing household relationships from the look of the household head as in previous research. This approach constitutes the first step in using more expanded categories of household’s relationships to understand child health by using more information about the household members such as age, sex, marital status and parental links in establishing their relationship with the reference child.

About policy implication, the analysis underscores the importance of interventions that target household level and other adults in a household besides biological parents. Further, programs should take into account the nature of the child’s health condition, whether it is long-term illness or short-term illness. Also, for health programs to reduce stunting and diarrhoea prevalence, they need to take into account the level of household socio-economic resources.

## Supplementary information

**Additional file 1.** Percent distribution of household member type, household wealth and age of the child, BFHS 2007

**Additional file 2.** Percent distribution of stunting and diarrhoea by household composition (Independent events), BFHS 2007

**Additional file 3.** Percent distribution of stunting and diarrhoea by household composition (Mutually exclusive), BFHS 2007

## Data Availability

The data analysed in this manuscript is from Statistics Botswana. The dataset is available by request from Statistics Botswana at http://www.statsbots.org.bw/. The dataset is also available from the author upon reasonable request and with permission from Statistics Botswana.

## Abbreviations

95% CI: 95% Confidence interval
BFHS: Botswana Family Health Survey
ChildId: Child identification number
Coef: Regression coefficient
Cons/cons: Residual variance at a particular level
DHS: Demographic Health Survey
EA: Enumeration area
HAZ: Height for age z- scores
HHId: Household identification number
HICs: High-income countries
LMICs: Low and middle-income countries
MQL1: Maximum quasi-likelihood
N: Number of children
n: Number of children in a sub-group
OR: Odds ratio
PCA: Principal component analysis
PQL2: The 2nd order predictive quasi-likelihood method
pr: probability
*p*-value: The observed significance level for the hypothesis test
ref.: Reference group
SD: Standard deviation
SE: Standard error
UNICEF: United Nations Children’s Fund
USA: United States of America
VPC: Variance partition coefficient
WHO: World Health Organization
χ^2^: Chi-squared test
