# Supplementary material for: Household composition and child health in Botswana
Source: BMC Public Health. 2019 Dec 3;19:1621. doi: 10.1186/s12889-019-7963-y (PMC6889653; doi:10.1186/s12889-019-7963-y)
Supplement: Supplementary file 1 — Additional file 1. Percent distribution of household member type, household wealth and age of the child, BFHS 2007 [file 12889_2019_7963_MOESM1_ESM.docx]

**Additional file 1:** Percent distribution of household member type, household wealth and age of the child, BFHS 2007

|  | Age group of the child | | | | | | | | | | | | | | | | | |
| --- | --- | --- | --- | --- | --- | --- | --- | --- | --- | --- | --- | --- | --- | --- | --- | --- | --- | --- |
|  | 0-5 months | | | 6-11 months | | | 12-23 months | | | 24-35 months | | | 36-47 months | | | 48-60 months | | |
| Characteristic | % | Total | 95%CI | % | Total | 95%CI | % | Total | 95%CI | % | Total | 95%CI | % | Total | 95%CI | % | Total | 95%CI |
| **Presence of household member** |  |  |  |  |  |  |  |  |  |  |  |  |  |  |  |  |  |  |
| Mother only | 10.8 | 73 | 8.6,13.4 | 13.1 | 89 | 10.6,16.1 | 21.5 | 146 | 18.4,25.0 | 19.7 | 134 | 16.8,23.0 | 18.6 | 127 | 15.8,21.9 | 16.3 | 111 | 13.6,19.5 |
| Father only | 2.9 | 11 | 1.6,5.2 | 6.6 | 25 | 4.5,9.8 | 23.6 | 88 | 19.2,28.5 | 20.7 | 78 | 16.8,25.2 | 25.8 | 97 | 21.3,30.9 | 20.3 | 76 | 16.2,25.2 |
| Both parents | 12.2 | 143 | 10.1,14.7 | 13.1 | 153 | 11.2,15.2 | 22.0 | 259 | 19.4,25.0 | 21.9 | 257 | 19.4,24.5 | 17.3 | 204 | 15.1,19.8 | 13.5 | 158 | 11.5,15.7 |
| No parents | 2.4 | 10 | 1.3,4.2 | 7.4 | 32 | 5.2,10.6 | 19.4 | 84 | 15.9,23.5 | 23.8 | 103 | 19.9,28.2 | 22.8 | 99 | 18.9,27.3 | 24.2 | 105 | 20.3,28.5 |
| Grandparent | 11.5 | 61 | 8.9,14.8 | 10.2 | 54 | 7.8,13.1 | 19.9 | 106 | 16.5,23.7 | 21.4 | 114 | 18.1,25.2 | 21.1 | 112 | 17.7,25.0 | 15.9 | 85 | 13.1,19.3 |
| Uncle | 11.5 | 95 | 9.3,14.1 | 10.5 | 86 | 8.5,12.8 | 21.9 | 181 | 19.1,25.0 | 22.0 | 181 | 19.1,25.1 | 19.0 | 157 | 16.3,22.0 | 15.2 | 125 | 12.8,18.0 |
| Aunt | 10.5 | 92 | 8.6,12.7 | 10.5 | 93 | 8.6,12.8 | 21.8 | 193 | 19.1,24.8 | 22.3 | 196 | 19.6,25.2 | 19.1 | 168 | 16.4,22.0 | 15.9 | 140 | 13.5,18.5 |
| Other relatives | 9.9 | 26 | 6.7,14.5 | 9.7 | 26 | 6.5,14.2 | 18.4 | 49 | 12.4,26.4 | 21.6 | 58 | 16.8,27.2 | 23.8 | 64 | 18.7,29.7 | 16.8 | 45 | 12.4,22.3 |
| Not related member | 9.9 | 24 | 4.7,19.5 | 10.2 | 25 | 6.6,15.5 | 20.2 | 49 | 15.3,26.2 | 25.3 | 62 | 19.8,31.8 | 15.4 | 38 | 11.3,20.7 | 19.0 | 46 | 14.1,25.1 |
| **Household wealth** |  |  |  |  |  |  |  |  |  |  |  |  |  |  |  |  |  |  |
| Poorest | 8.7 | 64 | 6.7,11.2 | 10.0 | 74 | 7.9,12.5 | 22.6 | 168 | 19.5,26.1 | 20.0 | 148 | 17.2,23.2 | 20.9 | 155 | 18.0,24.2 | 17.8 | 132 | 15.0,20.9 |
| Second | 10.3 | 79 | 18.3,12.6 | 11.0 | 85 | 9.0,13.5 | 20.5 | 158 | 17.7,23.6 | 21.5 | 165 | 18.6,24.5 | 19.7 | 152 | 17.0,22.8 | 17.1 | 132 | 14.5,20.1 |
| Middle | 7.0 | 30 | 4.9,9.9 | 12.9 | 55 | 9.7,16.9 | 22.0 | 95 | 18.2,26.4 | 22.1 | 95 | 18.3,26.6 | 16.5 | 71 | 13.1,20.5 | 19.5 | 84 | 15.7,23.8 |
| Fourth | 7.8 | 31 | 5.5,11.1 | 13.2 | 51 | 9.9,17.2 | 23.3 | 91 | 18.3,29.1 | 20.4 | 80 | 16.5,25.0 | 22.3 | 87 | 18.0,27.3 | 13.0 | 51 | 9.9,16.9 |
| Richest | 10.9 | 34 | 5.9,17.2 | 10.2 | 34 | 7.2,14.2 | 20.1 | 67 | 15.8,25.2 | 25.2 | 84 | 20.3,30.8 | 18.4 | 61 | 14.1,23.6 | 15.9 | 53 | 12.0,20.8 |
|  |  |  |  |  |  |  |  |  |  |  |  |  |  |  |  |  |  |  |
| **Grand Total** | 8.9 | 238 | 7.8,10.3 | 11.2 | 299 | 10.0,12.6 | 21.7 | 577 | 20.0,23.5 | 21.5 | 572 | 19.9,23.2 | 19.8 | 526 | 18.2,21.4 | 16.9 | 450 | 15.4,18.5 |

Motheronly=only the mother of the child is listed in the household, and not the father. Father only=only the father of the child is in the household, and not the mother.
