# Supplementary material for: Household composition and child health in Botswana
Source: BMC Public Health. 2019 Dec 3;19:1621. doi: 10.1186/s12889-019-7963-y (PMC6889653; doi:10.1186/s12889-019-7963-y)
Supplement: Supplementary file 2 — Additional file 2. Percent distribution of stunting and diarrhoea by household composition (Independent events), BFHS 2007 [file 12889_2019_7963_MOESM2_ESM.docx]

**Additional file 2:** Percent distribution of stunting and diarrhoea by household composition (Independent events), BFHS 2007

|  | Stunted (below -2 SD height/age) | | | | | Diarrhoea in the last two weeks before the survey | | | | |
| --- | --- | --- | --- | --- | --- | --- | --- | --- | --- | --- |
| Characteristic | % | Total | N | % miss. | 95% CI | % | Total | N | % miss. | 95% CI |
| **One parent (mother) present in household** |  |  |  |  |  |  |  |  |  |  |
| Mother+all | 30.4 | 631 | 680 | 7.2 | 26.8,34.4 | 15.5 | 680 | 680 | 0.0 | 12.8,18.5 |
| Mother+child only (2 persons) | 24.3 | 37 | 37 | 1.9 | 12.8,41.2 | 11.5 | 37 | 37 | 0.0 | 4.3,27.6 |
| Mother+grandparent+all | 36.4 | 95 | 99 | 4.2 | 27.1,46.8 | 17.6 | 99 | 99 | 0.0 | 11.0,26.9 |
| Mother+aunt+all | 29.3 | 216 | 234 | 7.6 | 23.0,39.4 | 16.4 | 234 | 234 | 0.0 | 12.0,22.0 |
| Mother+uncle+all | 40.4 | 62 | 64 | 3.8 | 27.7,54.5 | 16.7 | 64 | 64 | 0.0 | 9.2,28.4 |
| Mother+other relatives+not related+all | 26.7 | 114 | 122 | 6.4 | 18.7,36.6 | 17.2 | 122 | 122 | 0.0 | 11.2,25.5 |
| **One parent (father) present in household** |  |  |  |  |  |  |  |  |  |  |
| Father+all | 30.8 | 349 | 375 | 7.1 | 25.9,36.1 | 21.1 | 373 | 375 | 0.6 | 17.1,25.8 |
| Father+child only (2 persons) | 0.0 | 2 | 2 | 0.0 | 0.0 | 45.7 | 2 | 2 | 0.0 | 6.0,91.7 |
| Father+grandparent+all | 36.1 | 119 | 128 | 6.8 | 27.6,45.5 | 18.7 | 128 | 128 | 0.0 | 12.7,26.7 |
| Father+aunt+all | 29.4 | 154 | 166 | 7.4 | 22.3,37.7 | 22.1 | 164 | 166 | 1.2 | 16.1,29.5 |
| Father+uncle+all | 32.8 | 260 | 277 | 6.4 | 27.0,39.2 | 21.5 | 275 | 277 | 0.7 | 16.8,27.1 |
| Father+other relatives+not related+all | 31.4 | 49 | 52 | 6.8 | 19.6,46.4 | 9.9 | 52 | 52 | 0.0 | 4.4,20.9 |
| **Two parents present in household** |  |  |  |  |  |  |  |  |  |  |
| Both parents+all | 28.4 | 1093 | 1174 | 6.9 | 25,5,31.5 | 20.1 | 1174 | 1174 | 0.0 | 17.7,22.7 |
| Both parents+child only (3 persons) | 31.2 | 88 | 91 | 3.6 | 21.8,42.5 | 20.9 | 91 | 91 | 0.0 | 13.0,31.8 |
| Both parents+grandparent+all | 24.1 | 174 | 183 | 4.9 | 18.1,31.2 | 25.6 | 183 | 183 | 0.0 | 19.5,32.8 |
| Both parents+aunt+all | 28.5 | 327 | 341 | 4.2 | 23.7,33.9 | 20.6 | 341 | 341 | 0.0 | 16.4,25.4 |
| Both parents+uncle+all | 28.4 | 441 | 467 | 5.6 | 24.2,33.1 | 19.2 | 467 | 467 | 0.0 | 15.7,23.2 |
| Both parents+(other relatives+not related) +all | 29.4 | 217 | 230 | 6.0 | 21.7,38.5 | 14.7 | 230 | 230 | 0.0 | 10.3,20.4 |
| **No parents present in household** |  |  |  |  |  |  |  |  |  |  |
| No parents+all | 32.6 | 401 | 433 | 7.3 | 28.1,37.5 | 13.5 | 431 | 433 | 0.3 | 10.5,17.2 |
| No parents+ child (2 persons) | 22.3 | 13 | 13 | 0.0 | 6.7,53.2 | 4.5 | 13 | 13 | 0.0 | 0.6,27.1 |
| Grandparent +all | 25.3 | 117 | 122 | 3.6 | 18.1,34.2 | 17.7 | 122 | 122 | 0.0 | 11.8,25.7 |
| Aunt +all | 34.8 | 131 | 140 | 6.5 | 26.9,43.7 | 13.2 | 140 | 140 | 0.0 | 8.4,20.1 |
| Uncle+all | 32.4 | 17 | 17 | 2.7 | 14.6,57.5 | 15.9 | 17 | 17 | 0.0 | 3.8,47.4 |
| Other relatives+not related member +all | 32.8 | 74 | 77 | 3.8 | 23.2,44.2 | 11.3 | 77 | 77 | 0.0 | 5.9,20.7 |
|  |  |  |  |  |  |  |  |  |  |  |
| Grand Total | 29.9 | 2474 | 2662 | 7.1 | 28.0,31.9 | 18.0 | 2658 | 2662 | 0.1 | 16.5,19.6 |

The categories for household composition are defined from whether the child’s parents and other adult members are listed in the household. The categories in this table are independent events: they can occur at the same time. All refers to other household member, which is any of the combinations of grandparent/aunt/uncle/other relative/unrelated member. N=all children including those with missing data. Total=all children with and without stunting/diarrhoea .% miss=percentage missing.
