# Supplementary material for: Household composition and child health in Botswana
Source: BMC Public Health. 2019 Dec 3;19:1621. doi: 10.1186/s12889-019-7963-y (PMC6889653; doi:10.1186/s12889-019-7963-y)
Supplement: Supplementary file 3 — Additional file 3. Percent distribution of stunting and diarrhoea by household composition (Mutually exclusive), BFHS 2007 [file 12889_2019_7963_MOESM3_ESM.docx]

**Additional file 3:** Percent distribution of stunting and diarrhoea by household composition (Mutually exclusive), BFHS 2007

|  | Stunted (below -2 SD height/age) | | | | | Diarrhoea in the last two weeks before the survey | | | | |
| --- | --- | --- | --- | --- | --- | --- | --- | --- | --- | --- |
| Characteristic | % | Total | N | % miss. | 95% CI | % | Total | N | % miss. | 95% CI |
| **One parent (mother) present in household** | |  |  |  |  |  |  |  |  |  |
| Mother | 28.1 | 281 | 307 | 8.3 | 23.0,33.8 | 14.3 | 307 | 307 | 0.0 | 10.7,18.8 |
| Mother+grandparent | 61.4 | 30 | 32 | 6.1 | 43.4,76.8 | 10.3 | 32 | 32 | 0.0 | 3.8,24.9 |
| Mother+aunt | 29.5 | 125 | 137 | 8.5 | 21.8,38.5 | 15.9 | 234 | 137 | 0.0 | 10.5,23.4 |
| Mother +uncle | 49.3 | 17 | 17 | 0.0 | 26.0,72.9 | 20.3 | 17 | 17 | 0.0 | 7.3,45.1 |
| Mother +(other relatives+not related) | 21.9 | 61 | 63 | 3.3 | 12.3,35.8 | 16.9 | 63 | 63 | 0.0 | 8.9,29.7 |
| Multiple membership^a^ | 30.9 | 116 | 123 | 6.5 | 22.7,40.4 | 17.8 | 123 | 123 | 0.0 | 11.7,26.2 |
| **Total** |  |  | 679 |  |  |  |  | 679 |  |  |
| **One parent (father) present in household** |  |  |  |  |  |  |  |  |  |  |
| Father | 25.8 | 69 | 76 | 8.8 | 16.7,37.5 | 23.1 | 76 | 76 | 0.0 | 14.8,19.5 |
| Father+grandparent | 23.7 | 5 | 5 | 0.0 | 3.3,73.7 | 14.9 | 5 | 5 | 0.0 | 1.9,61.1 |
| Father+aunt | 3.6 | 5 | 6 | 13.3 | 0.4,25.5 | 13.3 | 6 | 6 | 0.0 | 1.7,57.8 |
| Father +uncle | 35.2 | 52 | 54 | 4.4 | 22.7,50.1 | 21.7 | 54 | 54 | 0.0 | 12.0,36.2 |
| Father+(other relatives+not related) | 29.1 | 10 | 12 | 13.8 | 8.3,65.0 | 6.0 | 12 | 12 | 0.0 | 0.8,34.7 |
| Multiple membership^a^ | 32.2 | 208 | 223 | 6.8 | 25.9,39.3 | 21.4 | 221 | 223 | 0.9 | 16.3,27.7 |
| **Total** |  |  | 375 |  |  |  |  | 375 |  |  |
| **Two parents present in household** |  |  |  |  |  |  |  |  |  |  |
| Both parents | 28.3 | 489 | 532 | 8.1 | 24.2,32.7 | 22.3 | 532 | 532 | 0.0 | 18.7,26.5 |
| Both parents+grandparent | 33.6 | 11 | 12 | 4.5 | 13.8,61.7 | 62.2 | 12 | 12 | 0.0 | 34.4,83.7 |
| Both parents+aunt | 29.3 | 20 | 22 | 7.4 | 13.0,65.5 | 15.8 | 22 | 22 | 0.0 | 5.2,39.1 |
| Both parents+uncle | 30.2 | 72 | 80 | 9.6 | 20.7,41.8 | 15.5 | 80 | 80 | 0.0 | 9.0,25.4 |
| Both parents+(other relatives+not related) | 29.0 | 125 | 134 | 7.0 | 17.9,43.2 | 10.5 | 134 | 134 | 0.0 | 6.1,17.6 |
| Multiple membership^a^ | 27.8 | 376 | 395 | 4.7 | 23.3,32.8 | 20.2 | 395 | 395 | 0.0 | 16.4,24.8 |
| **Total** |  |  | 1174 |  |  |  |  | 1174 |  |  |
| **No parents present in household** |  |  |  |  |  |  |  |  |  |  |
| No parents^b^ | 35.8 | 149 | 167 | 10.9 | 28.2,44.1 | 12.5 | 166 | 167 | 0.8 | 8.3,18.5 |
| Grandparent | 20.5 | 56 | 58 | 3.2 | 11.2,34.5 | 17.6 | 58 | 58 | 0.0 | 9.5,30.2 |
| Aunt | 39.4 | 77 | 84 | 7.9 | 28.8,51.1 | 12.8 | 84 | 84 | 0.0 | 6.9,22.5 |
| Uncle | 31.4 | 13 | 14 | 3.4 | 12.8,58.9 | 19.9 | 14 | 14 | 0.0 | 4.9,54.8 |
| Other relatives+not related member | 26.9 | 31 | 33 | 5.9 | 14.5,44.4 | 6.3 | 33 | 33 | 0.0 | 1.5,22.8 |
| Multiple membership^a^ | 31.1 | 75 | 77 | 3.3 | 21.7,42.4 | 15.3 | 77 | 77 | 0.0 | 8.9,25.0 |
| **Total** |  |  | 433 |  |  |  |  | 433 |  |  |
| Grand Total | 29.9 | 2474 | 2662 | 7.1 | 28.0,31.9 | 18.0 | 2658 | 2662 | 0.1 | 16.5,19.6 |

The categories for household composition are defined from whether the child’s parents and other adult members are listed in the household. The categories in this table are mutually exclusive: the groups can’t occur together. a=parent/no parent plus other persons.b=no parents in the household. N=all children including those with missing data. Total=all children with and without stunting/diarrhoea. % miss=percentage missing.
